# Intensive fasting reduces thrombosis and improves innate immunity

**DOI:** 10.18632/aging.204020

**Published:** 2022-04-16

**Authors:** Yanjun Yang, Jianrong Wang

**Affiliations:** 1 Suzhou Ninth Hospital Affiliated to Soochow University, Suzhou, 215200, China; 2 Research Center for Blood Engineering and Manufacturing, Cyrus Tang Medical Institute, Suzhou Medical College of Soochow University, Suzhou 215123, China

**Keywords:** intensive fasting, beego, thrombosis, innate immunity

Intensive fasting, known as beego in Chinese, is a complete water-only fasting format. To facilitate intensive fasting, psychological induction and mild physical exercise, such as meditation and breath control, are used to alleviate the feeling of hunger. To secure safety of intensive fasting, a gradual refeeding program is followed immediately after intensive fasting.

Light and moderate fastings belong to incomplete fasting, which includes calorie restriction, intermittent fasting, fasting-mimicking diets and periodic short-term water-only fasting with minimal calories or supplements. The incomplete fasting formulas appear to be more favored in Western countries, and have been intensively studied. For examples, incomplete fasting reduces inflammation [1], autoimmunity and multiple sclerosis symptoms [2], and optimizes immunological memory [3], blunts CD4 T helper cells activation and differentiation [4] and confers protection in CNS autoimmunity [5]. Incomplete fasting also affects immune cell dynamics and mucosal immune responses [6]. Nevertheless, incomplete fasting usually lasts a long duration until satisfactory outcomes are reached, often from weeks to months or even years, and compliance may not always be optimistic. Therefore, there is a need to consider intensive fasting format that does not last long for fasting period.

We recently performed a clinical study of intensive fasting comprising a complete fasting (7- and 14-day cohorts) and a 7-day refeeding program. Intensive fasting improved cardiovascular physiology, and selectively reduced blood pressure in hypertensive subjects; intensive fasting decreased blood triacylglycerol selectively in triacylglycerol-high subjects and increased cholesterol in all subjects during the fasting, but the cholesterol levels were normalized after completion of the refeeding program. Strikingly, intensive fasting reduced platelet production in those with normal levels of peripheral platelet count before fasting, and reduced platelet activation, aggregation, and degranulation, ultimately resulted in an alleviated thrombosis risk, yet maintained hemostasis by sustaining levels of coagulation factors and other hemostatic proteins. Downregulation of G6B and MYL9 may be involved in the regulation of the observed fasting-mediated reduction in platelets. These results together support that intensive fasting reduces thrombosis risk without compromising hemostasis capacity [7].

To explore whether shorter term intensive fasting is sufficient to impact on immune response, we applied multi-omics tools to analyze the CD45^+^ leukocytes from the subjects before and after 72-hour intensive fasting. Transcriptomic and proteomic profiling of CD45^+^ leukocytes revealed a predictive dynamic of extensive expression changes. Functional enrichment of differentially expressed genes and proteins revealed several pathways critically relevant to metabolic and immune cell functions. When focusing on specific leukocyte populations, peripheral neutrophils are distinctly increased by short-term intensive fasting. Proteomic analysis of leukocytes showed that short-term intensive fasting not only increased neutrophil degranulation, but also increased secretion of cytokines. Neutrophil cell viability was also increased by short-term intensive fasting. Our results suggest that short-term intensive fasting modulate immune function, in particular innate immune function, at least in part by influencing leukocytes expression profile [8]

As the first responders to bacterial infection, neutrophils exercise their bactericidal ability via mediating innate immune response, including phagocytosis, secretion of proteases, and release of neutrophil extracellular traps. Our transcriptomic and proteomic analysis of CD45^+^ cells revealed activated neutrophil function and neutrophil degranulation process, accompanied by increased granule contents, which suggests increased granule production. This degranulation process is a regulated exocytosis of secretory granules containing mediators such as proteases, lipases, and inflammatory mediators. In addition, cell migration and cell adhesion that are also related to neutrophil activation, were positively regulated in response to short-term intensive fasting. On the contrary, cytokines related to platelet activation are decreased. This is consistent with the reduction of thrombosis risk by intensive fasting [7].

Our study showed that intensive fasting has light or moderate side effects in clinical or observational studies [7,8], supporting that intensive fasting, particularly short-term intensive fasting can be implemented as noninvasive intervention for reducing thrombosis risk and modulating immune capacity.

## References

[1] Jordan S, et al. Cell. 2019; 178:1102–1114.e17. 10.1016/j.cell.2019.07.050 31442403 PMC7357241

[2] Choi IY, et al. Cell Rep. 2016; 15:2136–46. 10.1016/j.celrep.2016.05.009 27239035 PMC4899145

[3] Collins N, et al. Cell. 2019; 178:1088–1101.e15. 10.1016/j.cell.2019.07.049 31442402 PMC6818271

[4] Han K, et al. Nat Metab. 2021; 3:318–26. 10.1038/s42255-021-00356-0 33723462 PMC7990708

[5] Cignarella F, et al. Cell Metab. 2018; 27:1222–1235.e6. 10.1016/j.cmet.2018.05.006 29874567 PMC6460288

[6] Nagai M, et al. Cell. 2019; 178:1072–87.e14 10.1016/j.cell.2019.07.047 31442401

[7] Fang Y, et al. Prevention & Health. 2021; 4:4–17. 10.1136/bmjnph-2020-000183 34308107 PMC8258074

[8] Qian J, et al. Aging Cell. 2021; 20:e13507. 10.1111/acel.13507 34705313 PMC8590100

